# Telomerase Efficiently Elongates Highly Transcribing Telomeres in Human Cancer Cells

**DOI:** 10.1371/journal.pone.0035714

**Published:** 2012-04-27

**Authors:** Benjamin O. Farnung, Catherine M. Brun, Rajika Arora, Luca E. Lorenzi, Claus M. Azzalin

**Affiliations:** Institute of Biochemistry, Eidgenössische Technische Hochschule Zürich (ETHZ), Zürich, Switzerland; Tulane University Health Sciences Center, United States of America

## Abstract

RNA polymerase II transcribes the physical ends of linear eukaryotic chromosomes into a variety of long non-coding RNA molecules including telomeric repeat-containing RNA (TERRA). Since TERRA discovery, advances have been made in the characterization of TERRA biogenesis and regulation; on the contrary its associated functions remain elusive. Most of the biological roles so far proposed for TERRA are indeed based on *in vitro* experiments carried out using short TERRA-like RNA oligonucleotides. In particular, it has been suggested that TERRA inhibits telomerase activity. We have exploited two alternative cellular systems to test whether TERRA and/or telomere transcription influence telomerase-mediated telomere elongation in human cancer cells. In cells lacking the two DNA methyltransferases DNMT1 and DNMT3b, TERRA transcription and steady-state levels are greatly increased while telomerase is able to elongate telomeres normally. Similarly, telomerase can efficiently elongate transgenic inducible telomeres whose transcription has been experimentally augmented. Our data challenge the current hypothesis that TERRA functions as a general inhibitor of telomerase and suggest that telomere length homeostasis is maintained independently of TERRA and telomere transcription.

## Introduction

The physical ends of linear eukaryotic chromosomes are transcribed into a variety of non-coding RNA (ncRNA) species constituting the ‘telomeric transcriptome’. Among these species, the long ncRNA TERRA (telomeric repeat-containing RNA) was first discovered in mammalian cells and successively described in non-mammalian eukaryotes including zebrafish, *Arabidopsis thaliana*, the budding yeast *Saccharomyces cerevisiae* and the fission yeast *Schizosaccharomyces pombe* [1]–[7]. TERRA molecules are transcribed from the regions immediately preceding the telomeres -the subtelomeres- towards the end of the chromosomes primarily by the DNA-dependent RNA polymerase II (RNAPII), which uses the telomeric C-rich strand as a template. Hence, individual TERRA molecules comprise a subtelomeric tract and G-rich telomeric repeats (5′-UUAGGG-3′ in mammals). TERRA remains associated to telomeres post-transcriptionally suggesting that TERRA is a constitutive component of telomeric heterochromatin [1], [3], [4], [6]. Other RNA species transcribed from chromosome ends comprise ARIA, a C-rich telomeric RNA so far identified only in fission yeast and plants, and two complementary subtelomeric transcripts devoid of detectable telomeric repeats named ARRET, identified in budding and fission yeasts, and αARRET, identified only in fission yeast [1], [4], [5], [7].

Subtelomeric promoters driving the transcription of TERRA have been identified in human cells and comprise CpG dinucleotide-rich islands composed of stretches of 29 and 37 bp tandem repeats (29–37 repeats). 29–37 repeats are preceded by tandemly repeated 61 bp units that are dispensable for promoter activity and of so far uncharacterized function [8], [9]. CpG dinucleotides within TERRA promoters are heavily methylated in different cancer and primary cells [9]. In human colorectal carcinoma HCT116 cells knocked out for the two DNA methyltransferase enzymes DNMT1 and DNMT3b (DKO cells), TERRA promoter methylation is completely abolished and RNAPII binding to TERRA promoters and TERRA steady-state levels are markedly augmented [9]. Thus, the concerted activity of DNMT1 and 3b restricts TERRA promoter transcriptional activity, at least in HCT116 cells. Similarly, decreased subtelomeric CpG methylation is accompanied by increased TERRA cellular levels in cells derived from human patients affected by immunodeficiency, centromeric region instability, facial anomalies (ICF), a recessive syndrome deriving from germline mutations in the DNMT3b gene [10]. Strangely, TERRA abundance is reduced in mouse cells deficient for DNMT1 and DNMT3a/b, although global methylation of subtelomeres is compromised, suggesting species-specific mechanisms of TERRA regulation mediated by DNMTs [6], [11]. Indeed, TERRA promoters are still to be characterized in mouse cells and it remains possible that murine TERRA promoters are not regulated through CpG methylation.

While TERRA biogenesis and regulation have been extensively studied, a lack of reproducible experimental tools to alter TERRA cellular levels accounts for the sparse knowledge of TERRA-associated functions. Most of the putative roles so far ascribed to TERRA were deduced from *in vitro* experiments where short TERRA-like RNA oligonucleotides were employed. Such *in vitro* experiments have suggested that TERRA might regulate telomere length homeostasis, telomere replication and telomeric DNA condensation [6], [12]–[14]. In particular, TERRA-like oligonucleotides strongly inhibited telomerase activity in telomeric repeat amplification protocol (TRAP) and telomerase direct assays [6], [14]. Therefore, it is generally assumed that TERRA acts as a general inhibitor of telomerase-mediated telomere elongation and a few indirect *in vivo* evidences apparently support this assumption. A budding yeast telomere artificially forced to transcribe underwent shortening, while the length of the remaining telomeres was unaffected [15]. Yeast mutants with compromised Rat1p RNA exonuclease activity bear higher amounts of TERRA molecules and shorter telomeres as compared to wild type counterparts [16]. Finally, telomeres are shorter in cells established from ICF patients than in cells from healthy donors [10]. Nevertheless, it has not been directly tested whether the telomere shortening observed in these different cellular systems truly derives from telomerase inhibition. Thus, the actual biological relevance of the ability of TERRA-like oligonucleotides to inhibit telomerase activity *in vitro* still remains to be assessed.

In order to determine whether TERRA and telomere transcription affect telomerase activity *in vivo*, we have exploited two alternative human cellular systems where TERRA transcription is augmented. We show that DKO cells infected with retroviruses expressing the catalytic subunit of telomerase elongate their telomeres as efficiently as parental cells with intact DNMT activities. Consistently, telomerase biochemical activity appears not to be affected in the same cells. We also present the development of a cellular system where transcription of unique ‘transcriptionally inducible telomeres’ (tiTELs) can be controlled at will. Transcription induction of tiTELs does not affect their homeostatic length, nor does it prevent telomerase-mediated re-elongation upon shortening induced by telomerase inhibitors. Altogether our results challenge the commonly accepted notion that TERRA is a cellular inhibitor of telomerase and beg for exploring alternative functions exerted by TERRA and telomere transcription. In addition, our results imply that the telomere shortening observed in systems where TERRA was aberrantly elevated [10], [15], [16] likely derives from compromised telomere integrity rather than telomerase inhibition.

## Results and Discussion

### TERRA steady-state levels are maintained independently of telomere length in human cancer cells

The proposed role for TERRA in inhibiting telomerase activity has suggested a model where long telomeres accumulate or produce more TERRA, thereby preventing telomerase to further extend them [6], [14], [17]. Correlative evidence using non-isogenic mammalian cells lines of different origins seems to support this hypothesis [6]. On the contrary, recent work in budding yeast has shown that experimentally induced over-elongation or shortening of telomeres does not alter TERRA levels and RNAPII occupancy at chromosome ends [18]. We therefore set up to test whether a correlation exists between TERRA levels and telomere length in isogenic human cells with telomeres of different lengths.

**Figure 1:**
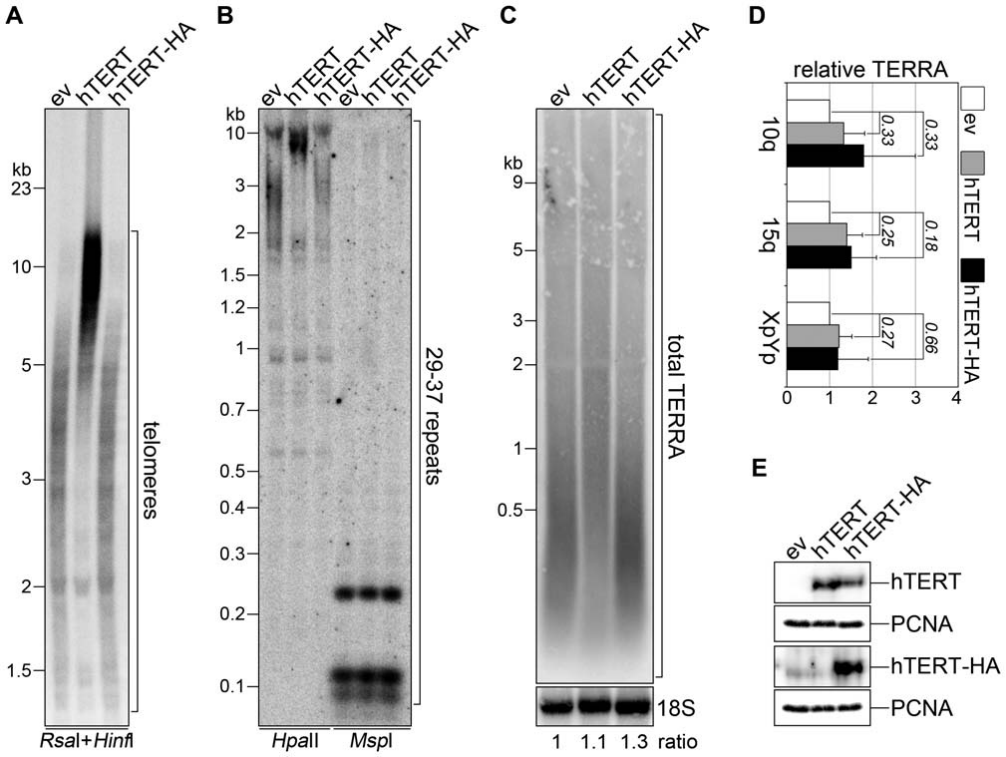
TERRA steady-state levels are not affected by telomere elongation in HeLa cells. (**A**) TRF analysis of HeLa cells infected with empty vector (ev), hTERT or hTERT-HA retroviruses. DNA was digested with *Rsa*I and *Hinf*I restriction enzymes and hybridized with telomeric probes. (**B**) The same DNA as in **A** was digested with *Hpa*II (methylation sensitive) or *Msp*I (methylation insensitive) restriction nucleases and hybridized with a probe detecting the 29–37 bp repeats of TERRA promoters. (**C**) Nuclear RNA was hybridized using telomeric probes to detect total TERRA and successively with 18S rRNA probes to control for loading. Numbers at the bottom are the ratios between TERRA and 18S signal expressed as fold increase over ev-infected samples. Molecular weights are on the left in kilobases. (**D**) qRT-PCR analysis of the steady-state levels of TERRA transcripts originating from 10q, 15q and Xp/Yp chromosome ends. Bars are averages from three independent experiments expressed as fold increase over ev-infected samples. Error bars and numbers are standard deviations and P-values, respectively. (**E**) Western blot analysis of infected cells using anti-hTERT (to detect all hTERT molecules), anti-HA (to detect hTERT-HA) and anti-PCNA (loading control) antibodies.

We stably infected cervical cancer HeLa cells with retroviruses expressing the catalytic subunit of human telomerase (hTERT). As a control, we infected the same cells with empty vector (ev) retroviruses or with retroviruses expressing hTERT C-terminally-fused to an influenza hemagglutinin epitope tag (hTERT-HA). hTERT-HA displays normal catalytic activity *in vitro* but fails to elongate telomeres *in vivo*, likely due to impaired recruitment to telomeres [19]. Stably infected populations were selected and maintained in culture for several population doublings (PDs). Telomere restriction fragment (TRF) analysis of genomic DNA indicated that at the time when the experiments were performed hTERT infection had led to a substantial elongation of bulk telomeres: TRFs were comprised between 2 and 5 kb in ev-infected cells and between 5 and more than 15 kb in hTERT-infected cells (Figure 1A). As anticipated, hTERT-HA expression did not alter telomere length, although it was expressed at levels comparable to hTERT (Figure 1A and E). We then digested genomic DNA with *Msp*I or with its CpG methylation sensitive isoschizomer *Hpa*II and hybridized it to radiolabelled probes corresponding to the 29–37 repeats found within TERRA promoters. Consistent with what we have previously reported [9], we found that 29–37 repeats are largely methylated in HeLa cells. The restriction pattern detected with 29–37 repeat probes was not altered in hTERT-expressing cells as compared to ev and hTERT-HA control cells, indicating that telomere elongation does not affect the methylation state of TERRA promoters (Figure 1B). Northern blot analysis of total RNA using telomeric probes did not disclose any substantial changes in total TERRA cellular levels in hTERT-infected cells as compared to ev- or hTERT-HA-infected controls (Figure 1C). A size shift of TERRA molecules towards higher molecular weights was evident in cells with elongated telomeres, suggesting that at least in these cell lines telomere length might influence the length of TERRA molecules (Figure 1C). Consistent with the northern blot analysis, quantitative real-time PCR (qRT-PCR) measurements of TERRA molecules transcribed from chromosome arms 10q, 15q and XpYp revealed no statistical significant change in TERRA steady-state levels upon telomere elongation (Figure 1D). We also performed analogous experiments in primary human lung fibroblasts (HLF) and, as for HeLa cells, telomere elongation did not significantly alter the methylation state of TERRA promoters and cellular TERRA levels (Figure S1).

**Figure 2:**
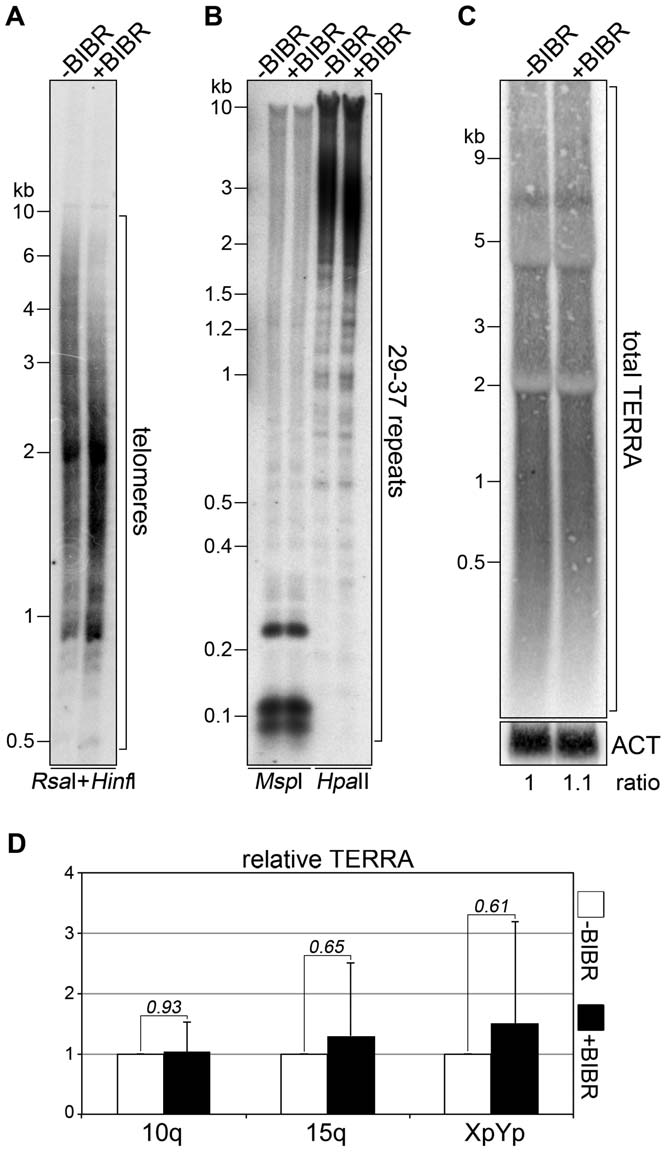
TERRA steady-state levels are not affected by telomere shortening in HeLa cells. (**A–D**) HeLa cells were treated with the telomerase inhibitor BIBR1532 (+BIBR) for 19 weeks or left untreated (−BIBR). Telomere length, TERRA promoter methylation and total or chromosome specific TERRA steady-state levels were analyzed as in Figure 1. Total RNA was used for all experiments. For northern blot analysis of total TERRA (**C**) beta-actin (ACT) was used as a normalization control. Molecular weights are on the left in kilobases.

In a complementary approach, we cultured HeLa cells in presence of the telomerase inhibitor BIBR1532 [20] for approximately 19 weeks. BIBR1532 treatment led to a substantial shortening of bulk telomeres: telomere length was mostly comprised between 2 and 5 kb in untreated control cells and between 1 and 3 kb in BIBR1532-treated cells (Figure 2A). Treated cells divided similarly to untreated cells indicating that telomeres were long enough to sustain normal cell growth (data not shown). Telomere shortening did not induce any substantial change in the methylation state of 29–37 repeats nor in total or chromosome specific TERRA steady-state levels (Figure 2B–D). Altogether, these experiments indicate that, as recently shown for budding yeast [18], telomere length alterations do not affect TERRA promoter methylation or TERRA cellular levels in human cells. We conclude that TERRA transcription is not regulated by telomere length, at least in the cell types that we have analyzed.

### DNA methyltransferase-deficient cells have normal telomerase activity

**Figure 3:**
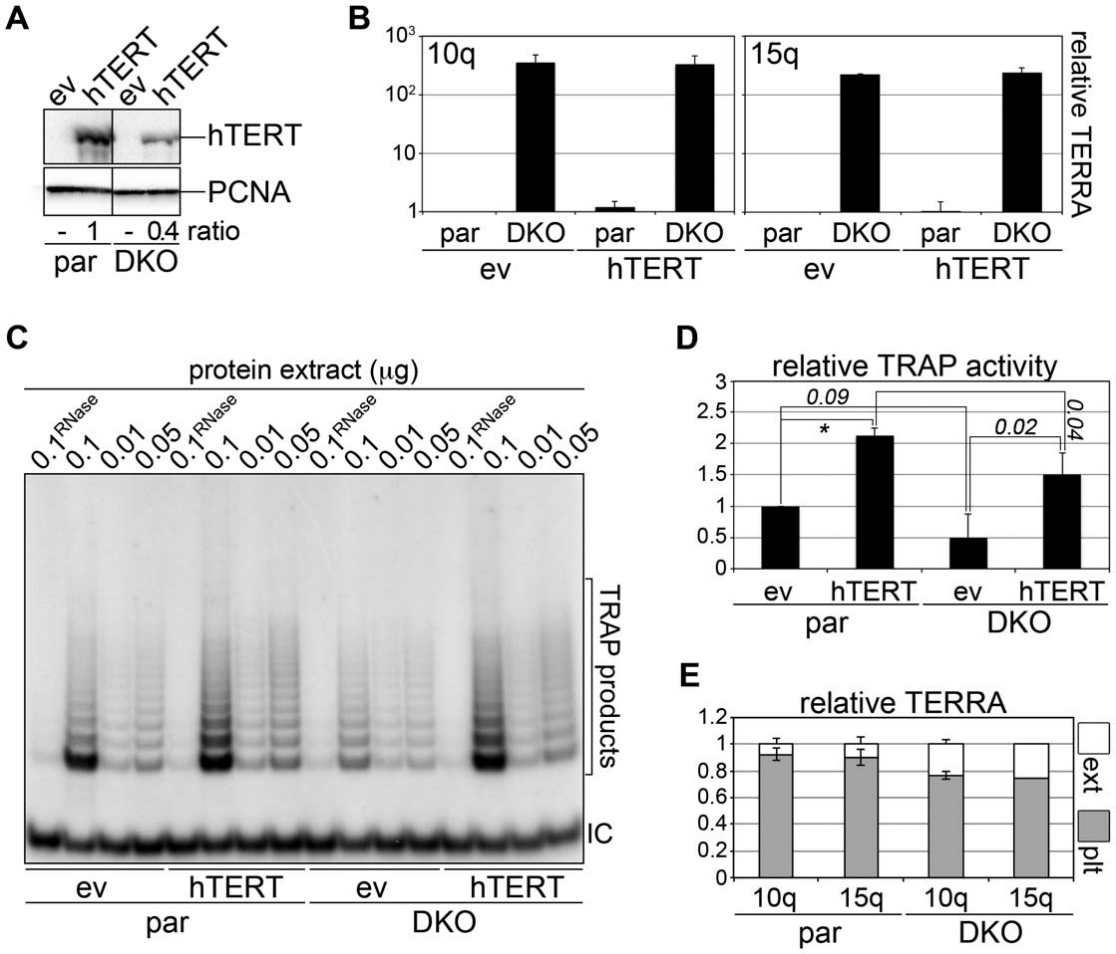
Cells deficient for DNMT1 and 3b display normal telomerase activity. (**A**) Western blot analysis of hTERT-expression in HCT116 parental (par) or DNMT1 and 3b double KO (DKO) infected cells. Numbers at the bottom are the ratios between hTERT and PCNA (loading control) signals, expressed as fold increase over hTERT-infected parental cells. (**B**) qRT-PCR analysis of the steady-state levels of TERRA transcripts originating from 10q and 15q chromosome ends expressed as fold increase over ev-infected par cells. Bars and error bars are averages and standard deviations from three independent experiments. (**C**) Analysis of TRAP amplification products from the indicated cells lines. Three different amounts of total proteins were used for each cell line and to control for specificity one sample was pre-treated with RNase A. Control primers amplifying an internal control (IC) were included in all reactions. (**D**) Quantification of telomerase activity in the indicated samples using qRT-PCR-based TRAP assays. Bars and error bars are averages and standard deviations from three independent experiments, after normalization through ev-infected parental cell samples. Numbers indicate P-values (*: P<0.01). (**E**) qRT-PCR-based quantification of 10q and 15q TERRA transcripts in TRAP extracts (ext) or in cell pellets left after extraction (plt). Relative TERRA amounts are expressed as fractions of total TERRA molecules from pellet plus extract. Bars and error bars are averages and standard deviations from three independent experiments.

The elevated TERRA levels present in DKO cells make them a suitable cellular system to test the effects exerted by TERRA on telomerase activity. We stably infected DKO and HCT116 parental (par) cells with hTERT or ev retroviruses and selected stable populations. Possibly due to the different transcriptional state of the two cell lines, we repeatedly found that over-expressed hTERT protein levels were more than two-fold higher in par than in DKO cells (Figure 3A). As shown by qRT-PCR and northern blot, the steady-state levels of TERRA molecules transcribed from 61-29-37 repeat-containing 10q and 15q chromosome ends were dramatically increased in DKO cells (Figures 3B and S2A) and, consistently with the results above described for HeLa and HLF cells, hTERT stable expression did not alter TERRA amounts in either cell line (Figure 3B). We prepared protein extracts and analyzed telomerase activity using quantitative TRAP assays. No significant difference in telomerase activity was measured in ev-infected par and DKO cells, while hTERT infection led to a substantial increase in telomerase activity in both cell lines (Figure 3C and D). TRAP activity was slightly higher in hTERT-infected par cells than in hTERT-infected DKO cells and we ascribe this difference to the lower amounts of total hTERT protein expressed in the DNMT-deficient cell line (Figure 3A, C and D). RNA isolated from TRAP extracts contained 10q and 15q TERRA molecules, although a large fraction of them (approximately 90 and 80% for par and DKO cells, respectively) remained in the cell pellets left after extraction (Figure 3E), likely because the majority of TERRA is chromatin associated [1], [3], [6]. Still, considering that 10q and 15q TERRA molecules are ∼300-fold more abundant in DKO than in par cells (Figure 3B), we estimate that DKO TRAP extracts contained ∼600-fold more TERRA molecules than par extracts. In conclusion, we report that TRAP activity is similar in cells with drastically different TERRA levels. This is in stark contrast with results obtained *in vitro* using short TERRA-like oligonucleotides [6], [14]. We believe that the short RNA oligonucleotides employed *in vitro* do not behave as natural, long TERRA molecules, possibly due to their use in high, non-physiological concentrations.

### Efficient telomerase-mediated telomere elongation in DKO cells

**Figure 4:**
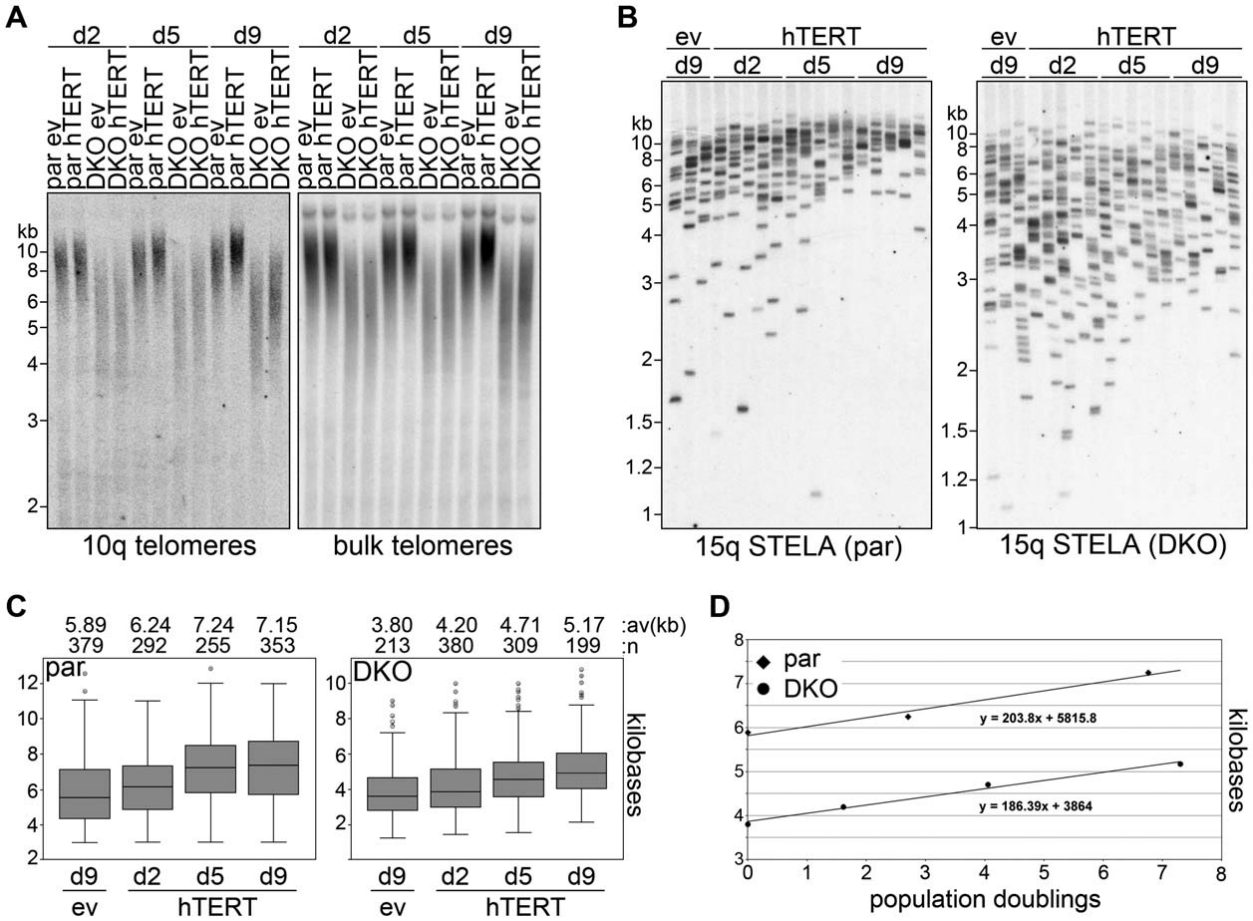
Efficient telomerase-mediated elongation of telomeres in DKO cells. (**A**) TRF analysis of par and DKO cells infected with empty vector (ev) or hTERT retroviruses. Genomic DNA was collected 2, 5 and 9 days (d) after infection, digested with *Rsa*I and transferred to a nylon membrane. The same membrane was first hybridized with a probe detecting 10q TRFs and successively with a probe detecting total telomeres. (**B**) STELA of 15q telomere length using the same DNA as in **A**. Marker molecular weights are on the left in kilobases. (**C**) Box plot representation of 15q telomere lengths. Average telomere lengths in kilobases and the number of total telomeres analyzed (n) are indicated for each sample. (**D**) 15q telomere elongation rates expressed as average telomere length at different population doublings. Note that for par cells, only three time points were used because no statistically significant difference in average telomere length was measured between day 5 and day 9.

To monitor how telomerase elongates telomeres in par and DKO cells we prepared genomic DNA from infected cells 2, 5 and 9 days after infection. We then performed TRF analysis using probes specifically detecting chromosome 10q subtelomeric sequences or telomeric repeat probes detecting bulk telomeres. Although 10q and bulk TRFs were substantially shorter in DKO than in parental cells, they both underwent progressive lengthening throughout the chosen time-course in cells infected with hTERT but not in ev control cells (Figure 4A). Thus, telomerase is able to elongate telomeres both in par and DKO cells including 10q telomeres, which are heavily transcribed in DKO cells (Figures 3B and S2A). To gain more quantitative data, we made use of single telomere length analysis (STELA), a PCR-based approach that allows to precisely measure the length of individual telomeres [21]. STELA of 15q telomeres confirmed that the average telomere length in DKO cells is lower than in parental cells (5.9 kb and 3.8 kb in par and DKO cells, respectively; Figure 4B and C). Nevertheless, as already suggested by TRF analysis, 15q telomeres were efficiently elongated in both cell lines infected with hTERT retroviruses (Figure 4D). By quantitative analysis of STELA products and normalization through the population doubling (PD) time calculated for the two cell lines (17.7 h and 29.6 h for par hTERT and DKO hTERT cells, respectively) we estimated that telomerase elongation rates were approximately ∼204 bp/PD in par cells and ∼186 bp/PD in DKO cells (Figure 4C and D). This slight difference in telomerase elongation rates does not correlate with the enormously increased levels of 15q TERRA in DKO cells (Figure 3B) and is likely to be ascribed to the different hTERT protein levels detected in infected par and DKO cells (Figure 3A). Trypan blue staining and fluorescence-activated cell sorter (FACS) analysis of Annexin V and propidium iodide-stained cells did not reveal any major difference in the fraction of dead or apoptotic cells in the different cell lines nor did it show substantial changes in the distribution of cells among the different phases of the cell cycle (Figure S2B). This indicates that the higher PD time measured for DKO cells is likely attributable to a longer cycling time of these cells, a notion further corroborated by the evident delay in cell cycle progression observed for synchronized DKO cells released from a nocodazole block (Figure S3A).

Normal telomere elongation in hTERT infected DKO cells does not support the notion that TERRA functions as a telomerase inhibitor *in vivo*. Still, we considered the possibility that TERRA transcripts in DKO cells are unable to inhibit telomerase either because they do not properly localize to telomeres or because they are drastically down-regulated during S-phase, when telomerase is acting [17], [22]. We combined indirect immunofluorescence with RNA fluorescence *in situ* hybridization (IF/RNA-FISH) to simultaneously detect the telomeric factor TRF2 and TERRA molecules. DKO cells contained more and brighter TERRA foci than parental cells and hTERT infections did not alter the overall pattern of TERRA hybridization. Approximately 20 and 30% of TRF2 foci co-localized with TERRA foci in par and DKO cells, respectively (Figure S2C and D), most likely reflecting the fact that the increased levels of TERRA in DKO cells facilitated the detection of TERRA foci. On the other hand, the fraction of TERRA foci co-localizing with telomeres was similar in both cellular backgrounds, indicating that the intrinsic ability of TERRA to remain associated to telomeric heterochromatin does not require intact DNMT activities (Figure S2C and D). Importantly, hTERT expression did not alter the co-localization rates in either cell line. Hence, the fraction of TERRA associated to telomeres is similar in par and DKO cells, and telomere elongation does not noticeably affect TERRA localization. We then blocked par and DKO cells in G2/M phase and released them synchronously into the cell cycle for a time sufficient to progress through S-phase (Figure S3A). We prepared total RNA at different time points and dot-blot hybridized it to TERRA and actin probes. TERRA levels remained stable throughout the entire time course in both cell lines (Figure S3B and C). Altogether, these observations disclose that telomerase-mediated telomere elongation is not substantially impaired in DKO cells, where TERRA transcription rates and steady-state levels are dramatically increased [9]. This lack of robust inhibition does not stem from mislocalization of TERRA or from its down-regulation during S-phase. This is well in line with our observations that telomerase activity is unaffected in the same cell lines.

### Generation of human cancer cell lines carrying transcriptionally inducible telomeres

**Figure 5:**
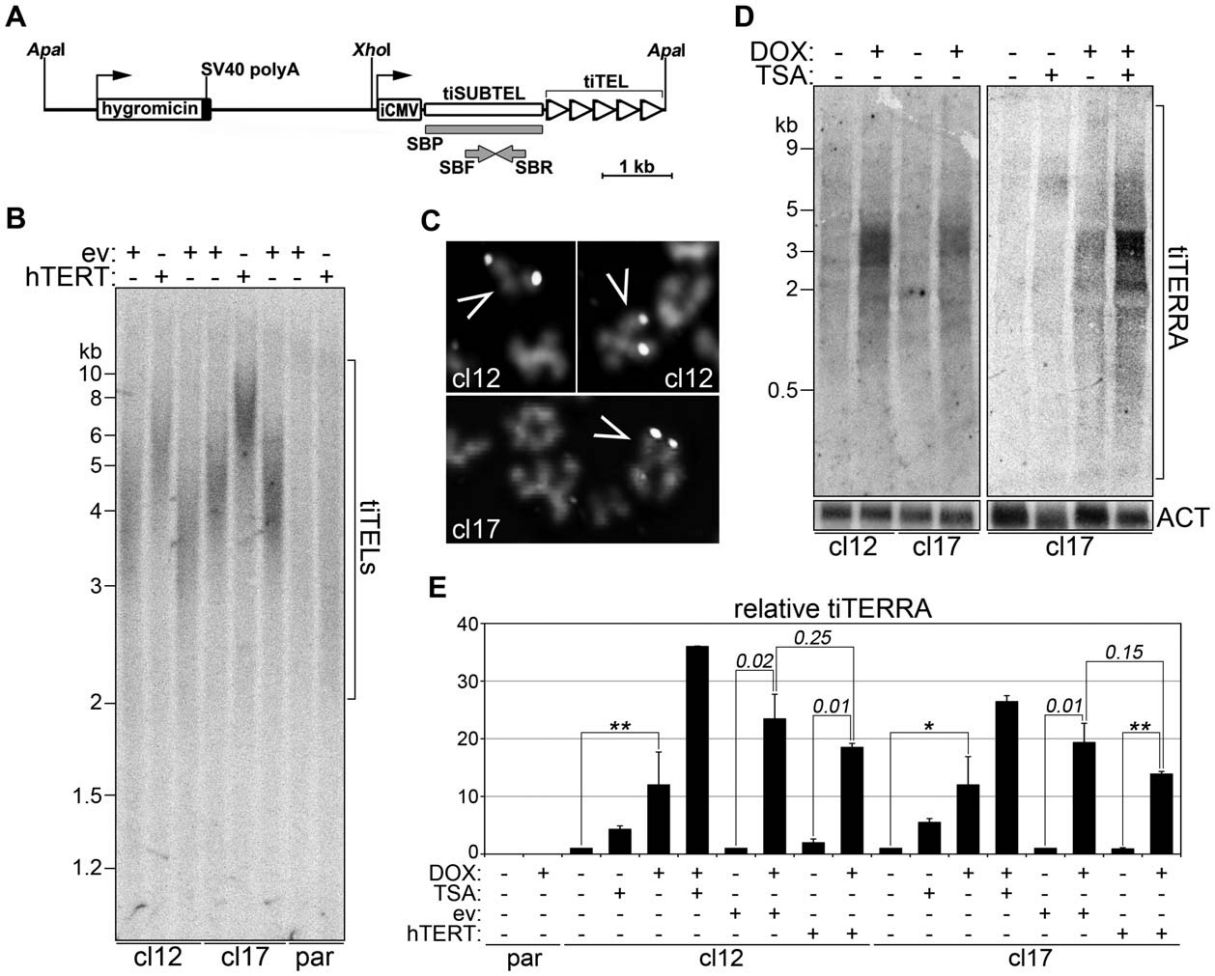
Generation and characterization of transcriptionally inducible telomeres (tiTELs). (**A**) Scheme of the tiTEL seeding vector. iCMV: inducible CMV promoter; SBF and SBR: oligonucleotides used in RT-PCR experiments; SBP: probe used in TRF, STELA and northern blot experiments. (**B**) tiTEL TRF analysis of genomic DNA prepared from clone 12 (cl12), clone 17 (cl17) and parental (par) cells infected with hTERT-expressing retroviruses or ev control retroviruses. DNA was hybridized using SBP probes. Marker molecular weights are on the left in kilobases. (**C**) Partial metaphases from cl12 and cl17 hybridized *in situ* to detect tiTELs (arrowheads). (**D**) Northern blot analysis of total RNA from cl12 and cl17 treated for 24 h with combinations of doxycycline (DOX) and trichostatin A (TSA) or left untreated. SBP probes were used to detect tiTERRA. The same membranes were stripped and re-probed to detect beta-actin transcripts (ACT) to control for loading. (**E**) qRT-PCR analysis of tiTERRA steady-levels in the indicated cell lines treated with different combinations of DOX and TSA or left untreated. For each cell line, values are expressed as fold increase over untreated samples. Bars and error bars are averages and standard deviations from 3 to 7 experiments. P values for relevant samples are indicated by numbers or by the asterisks (*: P<0.01; **: P<0.001).

DNMT1/3b deletion leads to global transcriptional changes in DKO cells [23]. To obtain a cellular system in which transcription of unique telomeres could be altered, we generated stable cell lines carrying transgenic telomeres that can be transcribed in an inducible fashion (‘transcriptionally inducible telomeres’; tiTELs). We combined the telomere seeding phenomenon with the inducible gene expression Tet-ON technology [24]–[26] and engineered a telomere seeding vector comprising a hygromycin resistance cassette and a doxycycline (DOX)-inducible minimal CMV promoter placed upstream of a (TTAGGG)n telomeric array (Figure 5A). A ∼1.6 kb unique sequence referred to as ‘transcriptionally inducible subtelomere’ (tiSUBTEL) separates the telomeric sequence from the inducible promoter (Figure 5A). The seeding plasmid was linearized by *Apa*I digestion in order to expose the telomeric tract at its 3′ end and transfected into a Tet repressor-expressing cell line derived from HeLa cells (T-Rex-HeLa). Independent hygromycin-resistant clones were selected and tested for tiTEL seeding by Southern blot analysis. Genomic DNA was digested with *Xho*I in order to release tiTEL TRFs and hybridized using radiolabeled probes corresponding to tiSUBTEL sequences (SBP in Figure 5A). Two clones (cl12 and cl17) showed the typical smearing hybridization pattern expected for newly seeded tiTELs (Figure 5B) and were chosen for further characterization. In both clones, tiTEL TRFs were mostly comprised between 3 and 6 kb. Because *Xho*I cuts approximately 2.4 kb upstream of the first telomeric repeat on the seeding plasmid (Figure 5A), tiTELs telomeric tracts were stabilized at approximately 0.6 to 3.4 kb, a size range comparable to the one of bulk telomeres in parental and in clonal cells (Figure S4A). We further confirmed tiTEL seeding in clones 12 and 17 using additional experimental approaches. We stably infected both clonal cell lines with hTERT-expressing retroviruses and confirmed that both tiTELs and natural telomeres were readily elongated upon hTERT infection (Figures 5B and S4A). STELA with oligonucleotides corresponding to tiSUBTEL sequences produced amplification products hybridizing to both SBP and telomeric probes only when we used genomic DNA from cl12 and cl17 cells but not from parental cells (Figure S4B). DNA FISH of metaphase chromosomes prepared from cl12 and cl17 cells using fluorescently labeled seeding plasmids devoid of telomeric repeats revealed that newly seeded tiTELs were integrated at one and two chromosome ends in clone 17 and in clone 12 cells, respectively (Figure 5C). Finally, dot blot hybridization of genomic DNA digested with the BAL31 exonuclease revealed a progressive disappearance of tiTEL hybridization signals over time (Figure S4C).

### Transcription induction of tiTELs

To test whether tiTELs responded to transcription induction, we cultured cl12 and cl17 cells in presence or absence of doxycycline (DOX) for 24 hours. We collected total RNA and subjected it to northern blot analysis using SBP probes (Figure 5A). DOX treatment led to the appearance of RNA species, referred to as ‘transcriptionally inducible TERRA’ (tiTERRA), comprised in length between approximately 0.5 and 6 kb (Figure 5D). Because the inducible promoter sequence is placed approximately 1.6 kb upstream of the telomeric sequence, we conclude that tiTEL transcription can proceed through most of the telomeric tract. We then performed qRT-PCR analysis by reverse transcribing RNA with random hexamers and PCR amplifying the obtained cDNA using SBF and SBR oligonucleotides (Figure 5A). As expected, no tiTERRA was detected in parental cells, confirming the specificity of the RT-PCR approach (Figure 5E). Although at low levels, tiTERRA transcripts were already detected in both uninduced clones, revealing a basal transcriptional activity from the inducible CMV promoter in absence of induction (Figure 5E). Absolute quantifications of tiTERRA molecules indicated that, in uninduced clones, tiTERRA was maintained at levels comparable to the ones of endogenous TERRA molecules transcribed from 10q chromosome ends (Figure S5A and B). This indicates that tiTERRA is maintained at physiological levels in both clonal cell lines. Consistent with the northern blot results, DOX treatment induced a 10-to-20-fold increase in tiTERRA levels in both clones (Figures 5E and S5B). TiTERRA molecules were also detected in PCR experiments performed with cDNA reverse transcribed using C-rich oligonucleotides (TelC) complementary to the telomeric stretch within TERRA molecules (Figure S5A and C). Thus, as with natural TERRA, individual tiTERRA transcripts contain both telomeric and subtelomeric sequences.

TERRA expression is epigenetically regulated and the histone deacetylase inhibitor trichostatin A (TSA) promotes accumulation of TERRA transcripts in human cancer cells [27]. We treated cl12 and cl17 cells with TSA in presence or absence of DOX and quantified tiTERRA by qRT-PCR. TSA alone already induced mild accumulation of tiTERRA molecules in both cell lines, while concomitant treatments with TSA and DOX led to a 25-to-38-fold increase in tiTERRA molecules as compared to untreated control cells (Figure 5E). Therefore, as with natural telomeres, tiTELs are embedded within heterochromatin and their transcription is repressed through mechanisms that require histone deacetylation. We also measured tiTERRA levels in cl12 and cl17 cells bearing elongated telomeres due to stable infection with hTERT retroviruses and found no statistically significant difference as compared to ev-infected control cells (Figure 5E). This indicates that telomere elongation does not influence tiTERRA expression and suggests that tiTEL transcription, similarly to the one of endogenous telomeres, is not controlled by canonical telomere position effect [28].

### Telomerase elongates tiTELs independently of tiTERRA induction

**Figure 6:**
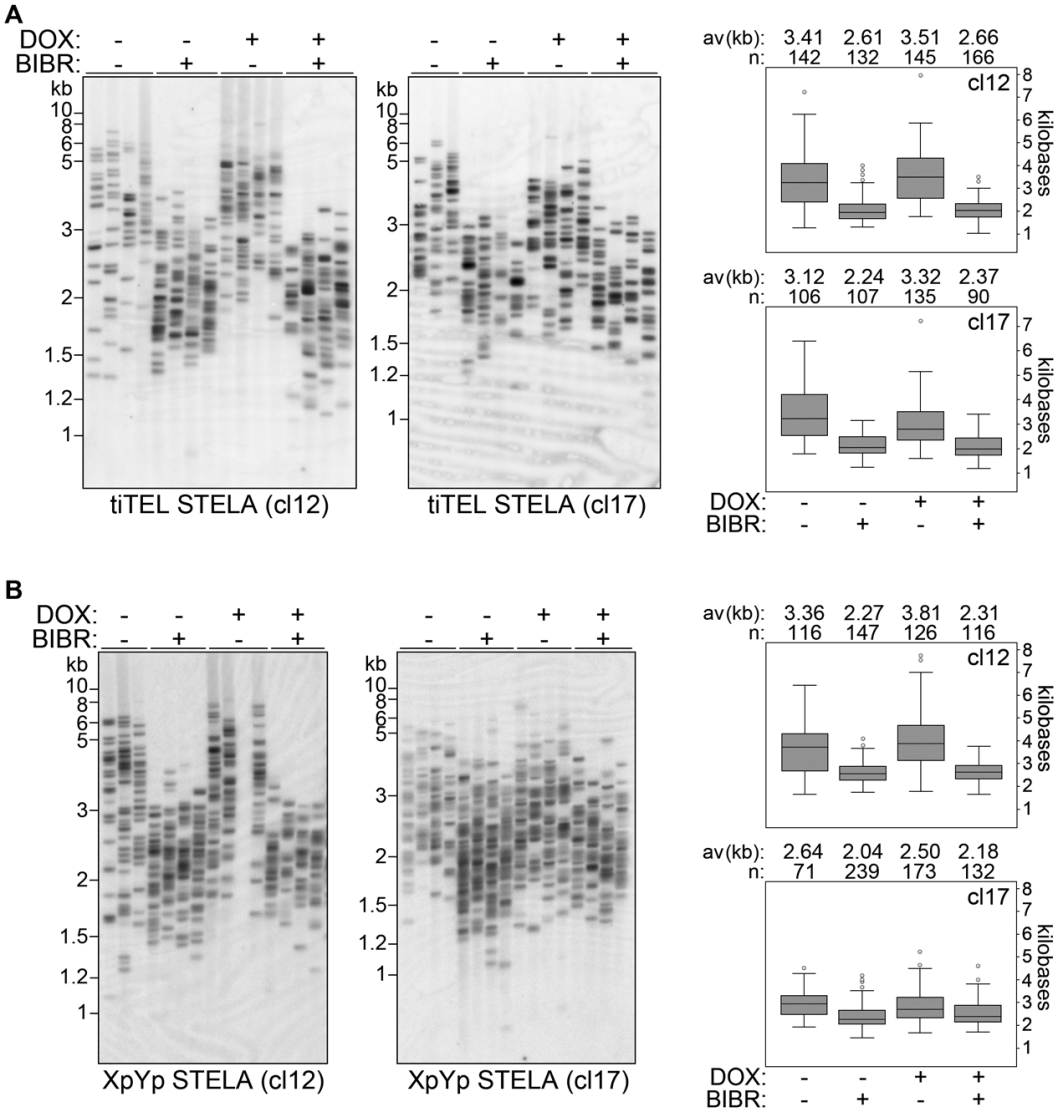
TiTEL length homeostasis is maintained upon prolonged tiTEL transcription induction. Cl12 and cl17 cells were treated with combinations of DOX and BIBR1532 (BIBR) for 60 population doublings. Genomic DNA was collected and STELA was performed for tiTELs (**A**) and XpYp telomeres (**B**). Marker molecular weights are on the left in kilobases. The Whisker-boxplots on the left represent STELA quantifications. Average (av) telomere lengths in kilobases and the number of total telomeres analyzed (n) are indicated for each sample.

To monitor whether tiTEL length homeostasis was affected by transcription induction, we cultured cl12 and cl17 cells in presence or absence of DOX for approximately 60 PDs. As a control we also treated cells with BIBR1532. Quantitative RT-PCR analysis performed at various time points during the course of the experiment confirmed that that tiTERRA induction was maintained in DOX-treated cells (data not shown). Moreover, the growth rates of DOX-treated and untreated cells were undistinguishable (data not shown). TiTEL STELA analysis revealed that DOX treatment did not induce any significant changes in tiTEL length as compared to untreated controls, while tiTEL shortening was readily detected in BIBR1532-treated cells as expected (Figure 6A). In addition, concomitant treatments with DOX and BIBR1532 shortened tiTELs to extents similar to the ones induced by BIBR1532 only (Figure 6A). Similar results were obtained when we analyzed the length of endogenous XpYp telomeres from the same cells (Figure 6B). These results indicate that tiTERRA transcription induction does not affect tiTEL length in a detectable manner, even upon prolonged treatments.

**Figure 7:**
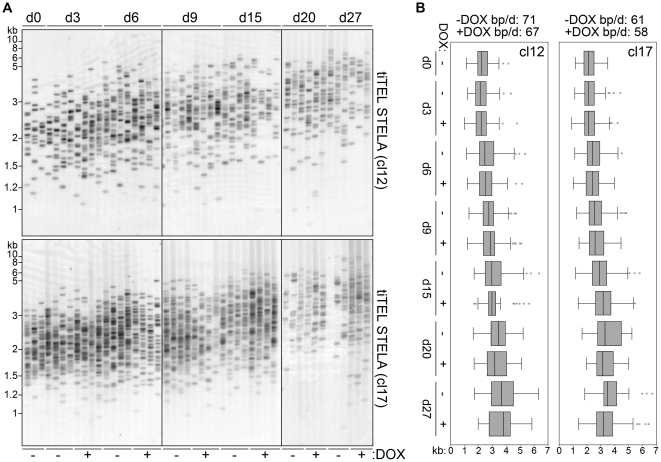
Telomerase elongates tiTELs independently of their transcription induction. (**A**) Cl12 and cl17 cells were treated with BIBR1532 for 60 population doublings and released into normal medium in presence or absence of DOX. STELA analysis of tiTELs was performed 0, 3, 6, 9, 15, 20 and 27 days (d) after release. Marker molecular weights are on the left in kilobases. (**B**) Whisker-boxplots represent STELA quantifications for each sample. Telomere elongation rates are expressed in base pairs per day (bp/d). All P-values between induced and uninduced samples were >0.1 and therefore are not indicated. For each condition, 77 to 400 telomeres were analyzed.

We reasoned that our analysis of tiTEL length homeostasis in cells treated with DOX could be biased by the fact that telomeres were already at their homeostatic length when we first induced tiTERRA transcription, while it is possible that TERRA-mediated inhibition of telomerase could only be revealed when telomerase is vigorously elongating telomeres. Therefore, we cultured cl12 and cl17 cells in presence of BIBR1532 for about 60 PDs in order to induce a substantial telomere shortening. At this point, no obvious impairment of cell division rates was observed indicating that telomeres were still long enough to sustain proper cell division (data not shown). We then washed off the telomerase inhibitor and cultured cells either in presence or absence of DOX over a time course of 27 days. STELA analysis revealed that tiTELs were re-elongated over the chosen time course with similar kinetics in DOX-treated and untreated samples. TiTEL elongation rates were: 67 and 71 bp/day for cl12 with and without DOX, respectively, P>0.1 for treated vs untreated samples at each analyzed time point; 58 and 61 bp/day for cl17 with and without DOX, respectively, P>0.1 (Figure 7A and B). We therefore conclude that tiTERRA transcription does not affect telomerase-dependent tiTEL lengthening in our clones. As previously explained for DKO cells, it remained possible that tiTERRA levels could be down-regulated during S-phase, when telomerase acts. Dot blot hybridization of RNA prepared from synchronized cl12 and cl17 cells confirmed that tiTERRA was maintained at higher levels in DOX-treated cells than in untreated controls during S-phase, thus disproving the above mentioned hypothesis (Figure S6). Our data clearly suggest that telomerase elongates tiTELs through pathways that are not regulated by tiTERRA or its transcription.

### Conclusions

By using independent yet complementary cellular systems, we have shown that telomerase-mediated telomere elongation is not substantially affected by the transcriptional activity associated to telomeres in human cancer cells. These observations argue against the currently accepted notion that TERRA might function as a general telomerase inhibitor, thereby contributing to maintain telomere length homeostasis. It still remains possible that TERRA could restrict telomerase activity in some particular contexts, for example in a specific set of tissues or during development. Our observations also indicate that the telomere shortening observed in cells with augmented TERRA levels, such as yeast cells carrying inducible telomeres, Rat1-deficient yeast cells, DKO cells and cells from ICF patients [4], [10], [15], is unlikely to derive from telomerase inhibition. One possible explanation is that the local increase of TERRA molecules at hyper-transcribing telomeres could harm their integrity for example by impairing replication. Supporting this model, human cancer cells depleted for the RNA surveillance factor UPF1 are characterized by aberrant accumulation of TERRA molecules at telomeric heterochromatin and sudden loss of telomeric sequences due to incomplete replication of the leading strand telomere [3], [29], [30]. Our data also highlight that the *in vitro* set up assembled using short TERRA-like oligonucleotides might not recapitulate accurately the biological scenario where TERRA is acting. Our tiTEL system could be used to carefully test to what extent the results so far obtained *in vitro* underscore the *in vivo* functions associated to TERRA and/or telomere transcription.

## Materials and Methods

### Cell culture procedures

HeLa, HEK293T, HLF, HCT116 par, HCT116 DKO and T-Rex-HeLa (Invitrogen) cells were cultured in high-glucose D-MEM (Gibco Life Technologies) supplemented with 10% of fetal calf serum or tetracycline-free fetal calf serum for T-Rex-HeLa cells, nonessential amino acids (Invitrogen), and penicillin/streptomycin (Invitrogen). Where indicated, 1 µg/ml doxycycline (Sigma-Aldrich), 200 ng/ml trichostatin A (Sigma-Aldrich), or 1 mM BIBR1532 (Tocris Bioscience) were added to the medium. Plasmid transfections were performed using the Lipofectamine 2000 reagent (Invitrogen) according to the manufacturer's instructions. Retroviruses were produced in HEK 293T cells according to standard procedures. Positively transfected or infected cells were selected in medium containing 200 mg/mL hygromycin (Fluka) or 1.5 µg/ml Puromycin (Sigma-Aldrich). For cell synchronizations, cl12 and cl17 cells were blocked in G1/S phase with 2 µg/ml aphidicolin (Sigma-Aldrich) for 20 h and released into fresh medium after washing them twice in 1× PBS. HCT116 par and DKO cells were blocked in G2/M phase with 60 ng/ml nocodazole (Sigma-Aldrich) for 14–18 h. Mitotic cells were collected by shake-off, washed three times in culture medium and released into fresh medium. Cell samples were collected at different time points after release and cell cycle progression was monitored by fluorescence-activated cell sorter (FACS) analysis of propidium iodide-stained cells using a BD FACSCalibur flow cytometer and the FlowJo software. To analyze cell viability, cells were stained with 0.2% trypan blue (Sigma) and counted using the Cellometer Auto T4 instrument (Nexcelom Bioscience). Apoptotic cells were scored using the Annexin V-FITC Apoptosis Detection Kit (eBioscience Diagnostics) according to manufacturers instructions.

### Plasmids

TiTEL seeding plasmids were constructed by inserting a 1.6-kb-long inverted luciferase cDNA downstream of the strong human cytomegalovirus (CMV) immediate-early promoter followed by two tetracycline operator sequences. A 1.2-kb-long (TTAGGG)n stretch was excised from the plasmid pCMVTelo (kind gift from Eric Gilson) and cloned immediately downstream of the Luciferase sequence. Retroviral plasmids pBABE-puro-hTERT and pBABE-puro-hTERT-HA were from Bob Weinberg's laboratory and were purchased from Addgene (plasmids 1771 and 1772). hTERT expression was monitored by standard western blot analysis of total protein extracts using rat monoclonal anti-hTERT antibodies for total hTERT (kind gift from Elena Giulotto), rabbit polyclonal anti-HA-tag antibodies for hTERT-HA (C29F4, Cell Signaling) and mouse monoclonal anti-PCNA antibodies to control for loading (sc-56, SantaCruz Biotechnology).

### Telomere length analysis

Total genomic DNA was collected by standard phenol/chloroform extraction or using the Wizard Genomic DNA Purification Kit (Promega). For telomere restriction fragment (TRF) analysis, 5 µg of genomic DNA were digested with *Rsa*I and/or *Hinf*I. For tiTEL TRF analysis, 15 µg of genomic DNA were digested with *Xho*I and *Ssp*I. For TERRA promoter methylation analysis, 10 µg of DNA were digested either with *Msp*I or *Hpa*II. Restriction fragments were electrophoresed in 0.7% (for TRF analysis) or 1.2% agarose gels (for methylation analysis) and transferred to nylon membranes (GE Osmonics). Alternatively, gels were dried for in gel hybridization. Hybridizations were performed for 16 h at 50–64°C using radiolableled DNA probes corresponding to a mixture of 1–5-kb-long telomeric DNA fragments (teloA probe, to detect bulk telomeres), to tiSUBTELs (SBP probe, to detect tiTELs), to 10q subtelomeric sequences (10q probe, to detect 10q TRFs), or to 29–37 repeats (29–37 probes, to detect TERRA promoter sequences). Post-hybridization washes were done at 50–64°C in 0.2–0.5× SSC, 0.2% SDS. Radioactive signals were detected using a Typhoon FLA 9000 phosphoimager (GE Healthcare). For STELA, 2 µg of DNA were digested with *Xho*I or *EcoR*I and purified with the GeneClean Turbo Kit (MP Biomedicals). 1 µl of 10 µM telorette 3 or telorette 4 oligonucleotides was mixed with 20 ng of digested DNA, incubated at 60°C for 10 min and cooled down to RT before adding 0.1 µl of 100 mM ATP, 0.1 µl of 400 U/µl T4 DNA ligase, 1 µl of 10× T4 DNA ligase buffer (New England Biolabs) and water up to 20 µl. Reactions were incubated at 35°C for 12 h and at 70°C for 15 min. 400 pg of ligated DNA were PCR amplified in 15 µl reactions containing 0.5 µM TelTail reverse primer, 0.5 µM forward primer (tiTEL-STELA, Xp/Yp-STELA, or 15qF), 0.3 mM dNTPs, 5 mM Tris-HCl (pH 8.8), 200 mM (NH_4_)_2_SO_4_, 0.01% Tween-20, 0.5 mM MgCl_2_, 1.5 U of Taq Thermoprime Polymerase (ABgene) and 0.15 U of Pwo polymerase (Roche). PCR cycling was as follows: 15 sec at 94°C, 30 sec at 65°C, 10 min at 68°C for 25 cycles. Amplified fragments were separated in 0.8% agarose gels and hybridized in gel using radiolabeled TeloA probe, SBP probe or a probe corresponding to 15q subtelomeres (15q probe). Hybridizations and signal detection were as for TRF analysis. For STELA analysis we used QuantityOne and R software. For each gel lane the fragment with the lowest intensity was considered as a single telomere and the corresponding signal was used to normalize all other fragments in the same lane. Molecular sizes were calculated using DNA molecular weight markers run in the same gels. Calculated telomere lengths were cumulated and used to compute average telomere length. Data were represented in Box-Whisker-plots showing the lowest value excluding outliers (data points that are less than 1.5 times the lower quartile range), the lower quartile, the median, the upper quartile and the highest value excluding outliers (data points that are more than 1.5 times the upper quartile). Outliers are represented by dots. For BAL31 experiments 20 µg of DNA were digested with 6 U of BAL31 (New England Biolabs) and 2 µg aliquots were collected at the indicated time points, dot blotted on nylon membranes and successively hybridized using SBP, TeloA and Alu repeat probes.

### RNA isolation and analysis

Total and nuclear RNA was prepared and digested with rDNase using the NucleoSpin RNAII Kit (Macherey-Nagel). RNA was additionally digested once for northern blot and dot blot analysis or twice for qRT-PCR analysis using DNaseI (Qiagen). For northern blotting, 10–15 µg of total or nuclear RNA were separated in 1.2% agrose formaldehyde gels and transferred to nylon membranes. For dot-blotting 1 µg of total RNA was spotted to nylon membranes. Membranes were hybridized with SBP, TeloA, 18S or β-actin radiolabeled probes as for genomic DNA hybridizations. Radioactive signals were detected using the Typhoon FLA 9000 phosphoimager and quantified using ImageJ or Quantity One software (BioRad). For qRT-PCR analysis 2–5 µg of total RNA were reverse-transcribed with Superscript II or III reverse transcriptases (Invitrogen) using random hexamers (New England Biolabs) or TeloC oligonucleotides and PCR amplified using the LightCycler 480 SYBR Green I Master mix (Roche) and the oligonucleotides listed in Table S1. PCR cycling was 10 sec at 98°C and 30 sec at 60°C for 45 cycles using the Rotor-Gene Q instrument (Qiagen). Averages, standard deviations, and P-values (two-tailed Student's t-test) were calculated with Microsoft Excel software. For absolute quantifications we generated standard curves using known amounts of plasmid DNA containing tiTEL or 10q subtelomeric sequences.

### Telomere repeat amplification protocol (TRAP) assays

For standard TRAP assays, 1×10^6^ cells were collected by trypsinization and lysed for 20 minutes on ice in TRAP extraction buffer (0.25 mM sodium deoxocholate, 1% Nonidet P-40, 150 mM NaCl, 10 mM Tris HCl pH 7.5, 1 mM MgCl_2_, 1 mM EGTA, 10% glycerol, 1 mM DTT, 1× protease inhibitor complex). Protein concentrations were determined using Bradford assays and 0.1, 0.05 and 0.01 µg of total proteins were used for TRAP reactions carried out as previously described [31]. Radioactive signals were detected and analyzed as above. qRT-PCR-based TRAP assays were performed using the Quantitative Telomerase Detection Kit (Allied Biotech Inc.) according to the manufacturer's instructions and the Rotor-Gene Q instrument (Qiagen).

### DNA and RNA fluorescence in situ hybridization (FISH)

DNA and RNA FISH experiments were carried out as previously described with a few modifications [24], [29], [32]. For DNA FISH, metaphase chromosome spreads were prepared from cl12 and cl17 cells and hybridized *in situ* using nick translation-labeled biotinylated probes corresponding to the entire seeding plasmid devoid of the telomeric tract. Before hybridization, chromosomes were treated with 10 µg/ml RNaseA for 30 min at 37°C and 0.005% pepsin in 0.01 N HCl for 20 minutes at 37°C. Chromosomes were then fixed in 4% paraformaldehyde diluted in 1× PBS. Each slide was hybridized with 300 ng of nick-translated probe. Slides were than washed three times in 50% formamide, 2× SSC at 42°C and three times in 2× SSC at 42°C. For probe detection, slides were washed in 4× SSC, 0.05% Tween-20, blocked in 5% milk in 4× SSC, 0.05% Tween-20 for 30 minutes at RT and incubated with 5 µg/ml avidin-fluoresceine (Vector Laboratories) in the same solution for 45 minutes at RT. Slides were washed three times in 4× SSC, 0.05% Tween-20 for 10 min at RT and then incubated with 5 µg/ml goat biotinylated anti-avidin antibodies (Vector Laboratories) for 45 min at RT. After three washes in 4× SSC, 0.05% Tween-20, we performed a second incubation with avidin-fluorescine for 45 minutes at RT and washes. DNA was stained with 4′,6-diamidino-2-phenylindole (DAPI) and images were acquired using the Deltavision multiplexed system (Applied Precision). For RNA FISH cells plated on coverslips were first incubated with mouse monoclonal anti-human TRF2 primary antibodies (4A794, Millipore) and successively with donkey anti-mouse secondary antibody conjugated with Alexa 488 (Invitrogen). Cells were fixed in 4% paraformaldehyde and subjected to RNA FISH using TeloA probes labeled with Cy3-dCTP (Perkin Elmer). Images were taken as 0.2 µm Z-stacks using the Deltavision Multiplexed system (Applied Precision). TRF2 and TERRA foci were analyzed using the spot detection and co-localization applications of the Imaris software (Bitplane). Foci were estimated to have an average diameter of 0.7 µm and they were considered to be co-localizing when found to be 0.6 µm or less apart from each other.

## Supporting Information

Figure S1 **Shows that TERRA steady-state levels are not affected by telomere elongation in human primary fibroblasts.** (**A**) TRF analysis of human lung primary fibroblasts (HLF) infected with empty vector (ev), hTERT or hTERT-HA retroviruses. DNA was digested with *Rsa*I and *Hinf*I restriction enzymes and hybridized with telomeric probes. (**B**) The same DNA as in **A** was digested with *Hpa*II (methylation sensitive) or *Msp*I (methylation insensitive) restriction nucleases and hybridized with a probe detecting the 29–37 bp repeats of TERRA promoters. (**C**) Total RNA was hybridized using telomeric probes to detect total TERRA and successively with beta-actin (ACT) probes to control for loading. Numbers at the bottom are the ratios between TERRA and actin signals expressed as fold increase over ev-infected samples. Molecular weights are on the left in kilobases. (**D**) qRT-PCR analysis of the steady-state levels of TERRA transcripts originating from 10q, 15q and Xp/Yp chromosome ends. Bars are averages from three independent experiments expressed as fold increase over ev-infected samples. Error bars and numbers are standard deviations and P-values, respectively. *: P<0.01. (**E**) Western blot analysis of infected cells using anti-hTERT (to detect all hTERT molecules), anti-HA (to detect hTERT-HA) and anti-PCNA (loading control) antibodies.
(TIF)

Figure S2 **Shows 10q TERRA steady-state levels, cellular state and TERRA localization in par and DKO cells upon telomere elongation.** (**A**) Total RNA was hybridized using probes to detect TERRA molecules transcribed from 10q subtelomeres in the indicated cell lines. The same membrane was stripped and hybridized with 18S rRNA probes to control for loading. Molecular weights are on the left in kilobases. (**B**) Top: examples of FACS analysis of Annexin V and propidium iodide stained cells. Bottom: quantifications of alive cells (trypan blue negative cells), apoptotic cells (Annexin V – AV – positive cells) and cells in the different phases of the cell cycle as judged by propidium iodide (PI) staining. Values are averages and standard deviations form three independent experiments. (**C**) Examples of anti-TRF2 indirect immunofluorescence combined with TERRA RNA FISH in the par and DKO cells infected with hTERT or empty vector (ev) retroviruses. In the merge panels TRF2 is in green, TERRA in red and DAPI-stained DNA in blue. (**D**) Quantification of co-localization of TRF2 and TERRA foci. Bars and error bars are averages and standard deviations of co-localization events per nucleus. For each condition 50 nuclei were analyzed.
(TIF)

Figure S3 **Shows TERRA steady-state levels in synchronized par and DKO cells.** (**A**) FACS profiles of propidium iodine-stained cells blocked in G2/M using nocodazole and released into the cell cycle for the indicated hours. (**B**) Dot-blot analysis of total RNA isolated at indicated hours after release. The same membranes were first hybridized with telomeric probes (to detect TERRA), stripped and re-hybridized with beta-actin probes to control for loading. (**C**) Quantification of dot blots as in **B**. TERRA values were normalized through the corresponding actin values and expressed as fold increase over par cells at time 0. Bars and error bars represent averages and standard deviations from two independent experiments.
(TIF)

Figure S4 **Shows the characterization of tiTELs in cl12 and cl17 cells.** (**A**) TRF analysis of bulk telomeres using *Hinf*I and *Rsa*I-digested DNA hybridized to telomeric probes. (**B**) Control experiments demonstrating specificity of tiTEL STELAs. Nt: no template control. The same membrane was first hybridized using SBP probes, stripped and re-hybridized using telomeric probes. (**C**) Dot blot hybridization of BAL31-digested genomic DNA from parental (par), cl12 and cl17 cells. The same membrane was hybridized successively to detect tiTEL, telomeric repeat and alu repeat DNA. Radioactive signals associated to the three hybridizations are quantified below. Note that alu signals remain constant throughout the time course, while telomeric and tiTEL signals gradually diminish in both clonal cell lines.
(TIF)

Figure S5 **Shows the characterization of tiTEL transcription induction in cl12 and cl17 cells.** (**A**) Sketch of oligonucleotides used in RT-PCR experiments. TelC oligonucleotides were used for RT, SBF and SBR oligonucleotides were used for PCR. (**B**) Absolute quantification of tiTERRA (ti) and natural TERRA transcribed from 10q chromosome ends in cl12 and cl17 cells treated or not with DOX. (**C**) Agarose gel analysis of tiTERRA RT-PCR products from cl12 and cl17 cells treated or not with DOX.
(TIF)

Figure S6 **Shows tiTERRA and total TERRA steady-state levels in synchronized cl12 and cl17 cells.** (**A**) FACS profiles of propidium iodine-stained cells blocked in G1/S using aphidicolin and released into the cell cycle for the indicated hours in presence or absence of DOX. (**B**) Dot blot analysis of total RNA isolated at indicated hours after release. The same membranes were hybridized successively with SBP (to detect tiTERRA), telomeric (to detect total TERRA) and beta-actin (loading control) probes. (**C**) Quantification of dot-blots as in **B**. TERRA values were normalized through the corresponding actin values and expressed as fold increase over unsynchronized, untreated cells. Points and error bars represent averages and standard deviations from two independent experiments.
(TIF)

Table S1 **Shows the oligonucleotides used in this study.**
(DOCX)
