# Supplementary figures and images for: Telomerase Efficiently Elongates Highly Transcribing Telomeres in Human Cancer Cells
Source: PLoS One. 2012 Apr 27;7(4):e35714. doi: 10.1371/journal.pone.0035714 (PMC3338753; doi:10.1371/journal.pone.0035714)

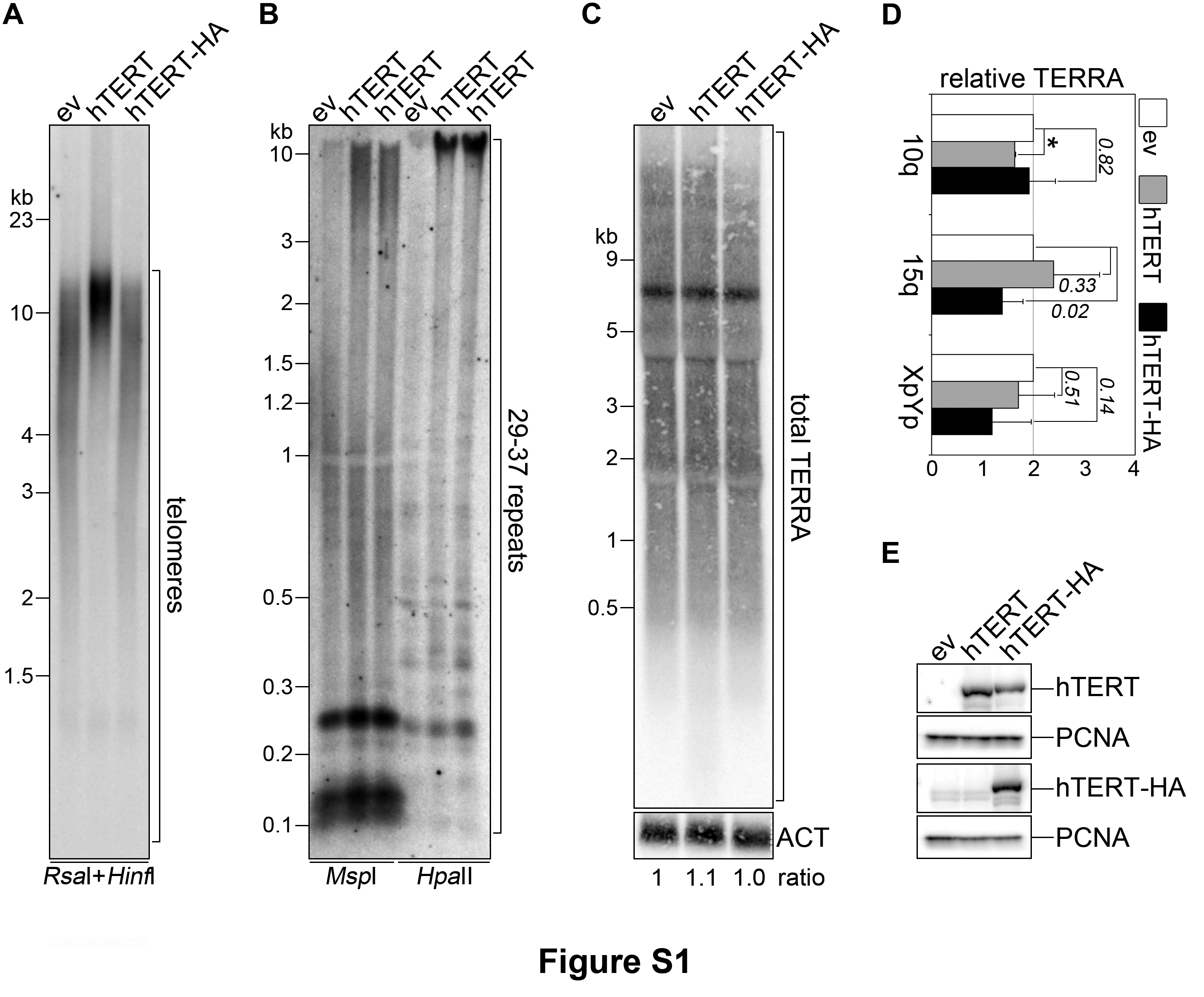

Supplement: Figure S1 — Shows that TERRA steady-state levels are not affected by telomere elongation in human primary fibroblasts. (A) TRF analysis of human lung primary fibroblasts (HLF) infected with empty vector (ev), hTERT or hTERT-HA retroviruses. DNA was digested with RsaI and HinfI restriction enzymes and hybridized with telomeric probes. (B) The same DNA as in A was digested with HpaII (methylation sensitive) or MspI (methylation insensitive) restriction nucleases and hybridized with a probe detecting the 29–37 bp repeats of TERRA promoters. (C) Total RNA was hybridized using telomeric probes to detect total TERRA and successively with beta-actin (ACT) probes to control for loading. Numbers at the bottom are the ratios between TERRA and actin signals expressed as fold increase over ev-infected samples. Molecular weights are on the left in kilobases. (D) qRT-PCR analysis of the steady-state levels of TERRA transcripts originating from 10q, 15q and Xp/Yp chromosome ends. Bars are averages from three independent experiments expressed as fold increase over ev-infected samples. Error bars and numbers are standard deviations and P-values, respectively. *: P<0.01. (E) Western blot analysis of infected cells using anti-hTERT (to detect all hTERT molecules), anti-HA (to detect hTERT-HA) and anti-PCNA (loading control) antibodies. (TIF) [file pone.0035714.s001.tif]

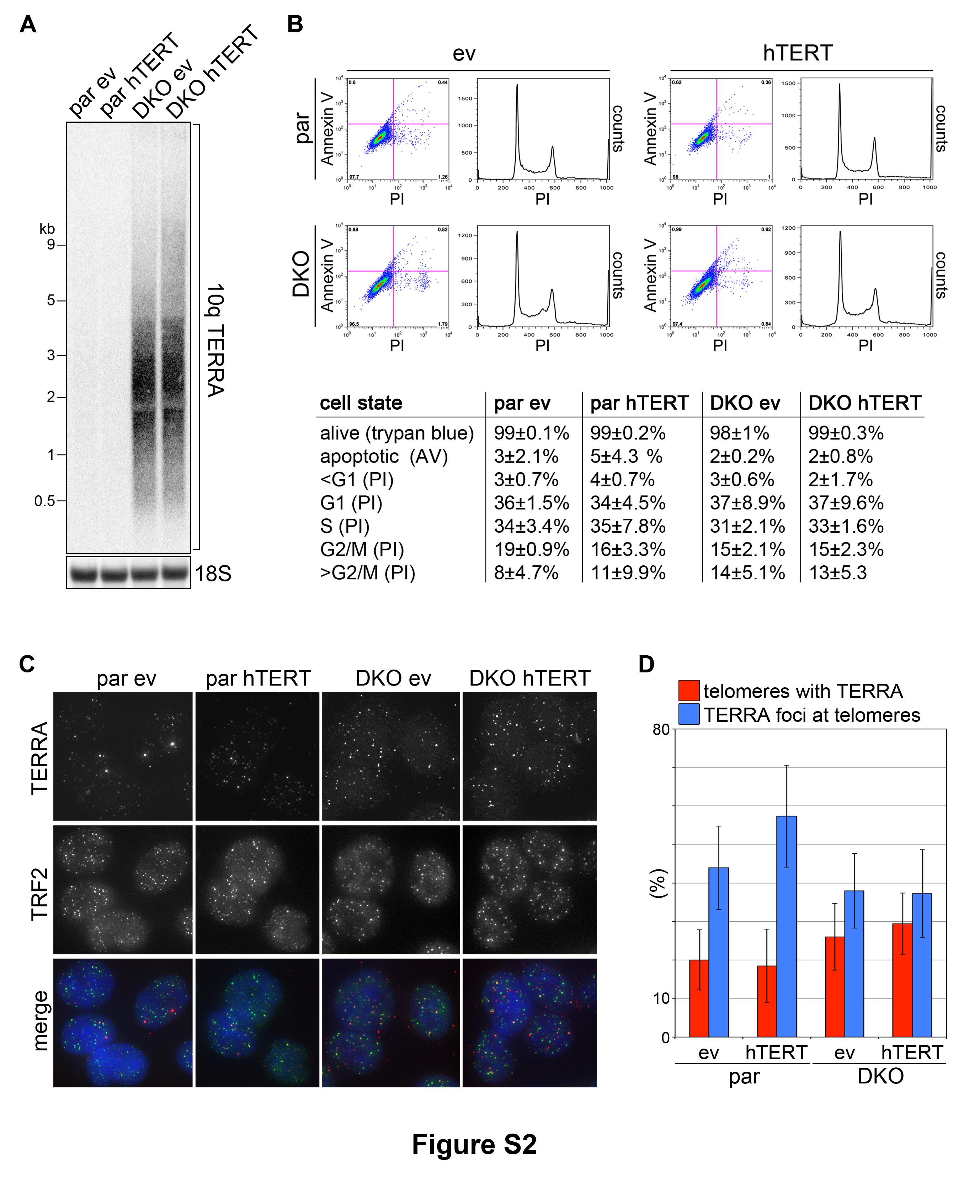

Supplement: Figure S2 — Shows 10q TERRA steady-state levels, cellular state and TERRA localization in par and DKO cells upon telomere elongation. (A) Total RNA was hybridized using probes to detect TERRA molecules transcribed from 10q subtelomeres in the indicated cell lines. The same membrane was stripped and hybridized with 18S rRNA probes to control for loading. Molecular weights are on the left in kilobases. (B) Top: examples of FACS analysis of Annexin V and propidium iodide stained cells. Bottom: quantifications of alive cells (trypan blue negative cells), apoptotic cells (Annexin V – AV – positive cells) and cells in the different phases of the cell cycle as judged by propidium iodide (PI) staining. Values are averages and standard deviations form three independent experiments. (C) Examples of anti-TRF2 indirect immunofluorescence combined with TERRA RNA FISH in the par and DKO cells infected with hTERT or empty vector (ev) retroviruses. In the merge panels TRF2 is in green, TERRA in red and DAPI-stained DNA in blue. (D) Quantification of co-localization of TRF2 and TERRA foci. Bars and error bars are averages and standard deviations of co-localization events per nucleus. For each condition 50 nuclei were analyzed. (TIF) [file pone.0035714.s002.tif]

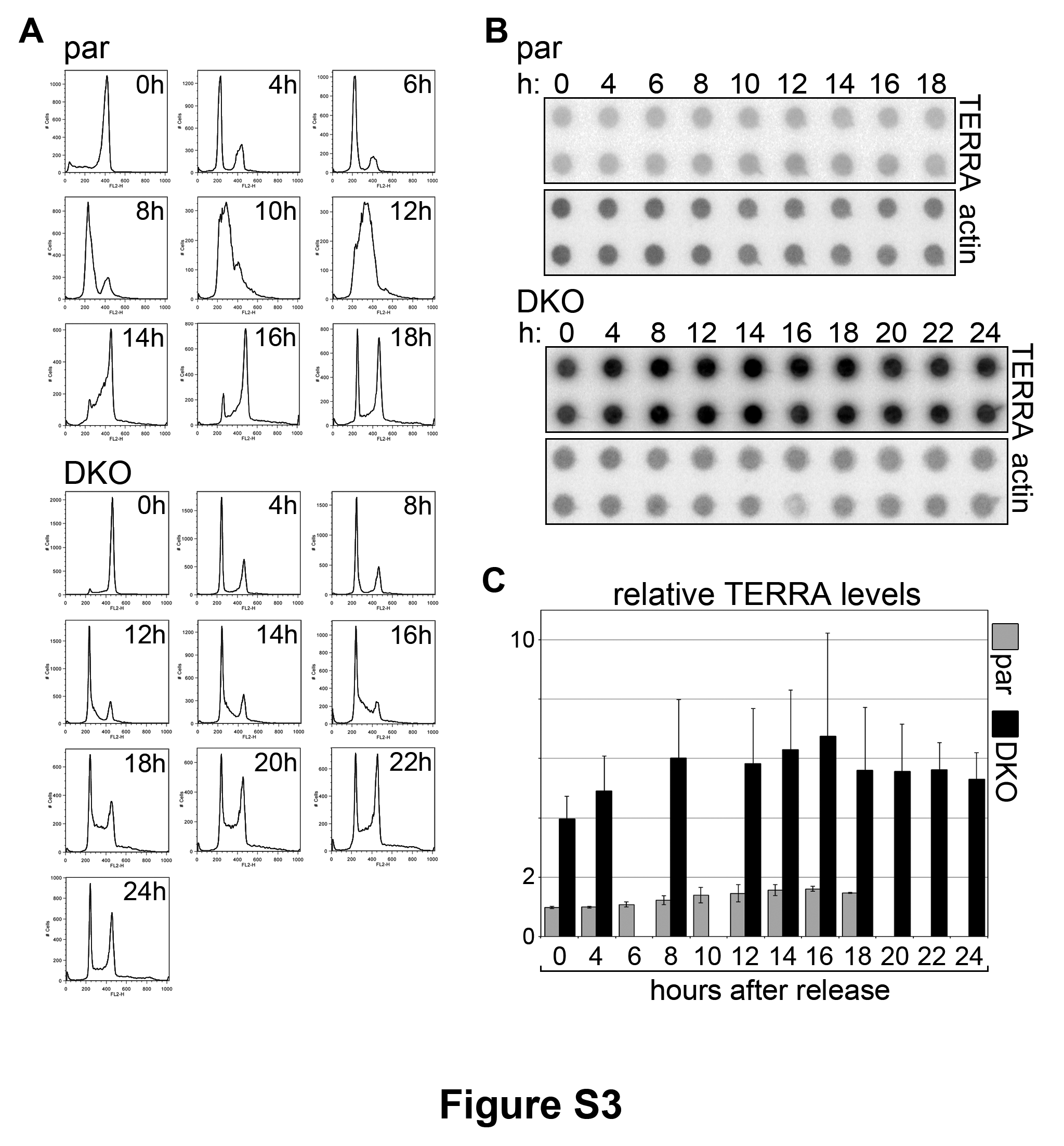

Supplement: Figure S3 — Shows TERRA steady-state levels in synchronized par and DKO cells. (A) FACS profiles of propidium iodine-stained cells blocked in G2/M using nocodazole and released into the cell cycle for the indicated hours. (B) Dot-blot analysis of total RNA isolated at indicated hours after release. The same membranes were first hybridized with telomeric probes (to detect TERRA), stripped and re-hybridized with beta-actin probes to control for loading. (C) Quantification of dot blots as in B. TERRA values were normalized through the corresponding actin values and expressed as fold increase over par cells at time 0. Bars and error bars represent averages and standard deviations from two independent experiments. (TIF) [file pone.0035714.s003.tif]

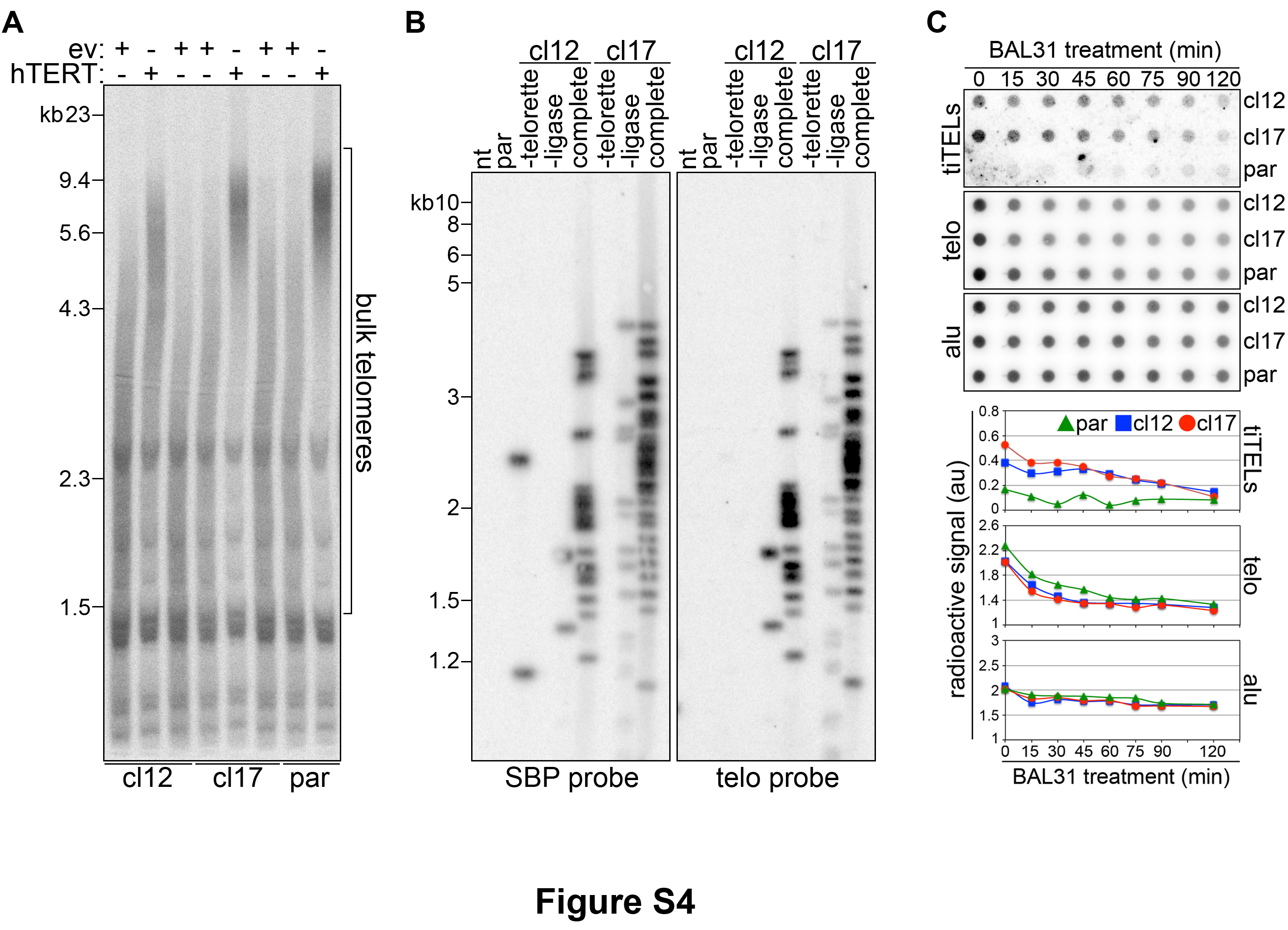

Supplement: Figure S4 — Shows the characterization of tiTELs in cl12 and cl17 cells. (A) TRF analysis of bulk telomeres using HinfI and RsaI-digested DNA hybridized to telomeric probes. (B) Control experiments demonstrating specificity of tiTEL STELAs. Nt: no template control. The same membrane was first hybridized using SBP probes, stripped and re-hybridized using telomeric probes. (C) Dot blot hybridization of BAL31-digested genomic DNA from parental (par), cl12 and cl17 cells. The same membrane was hybridized successively to detect tiTEL, telomeric repeat and alu repeat DNA. Radioactive signals associated to the three hybridizations are quantified below. Note that alu signals remain constant throughout the time course, while telomeric and tiTEL signals gradually diminish in both clonal cell lines. (TIF) [file pone.0035714.s004.tif]

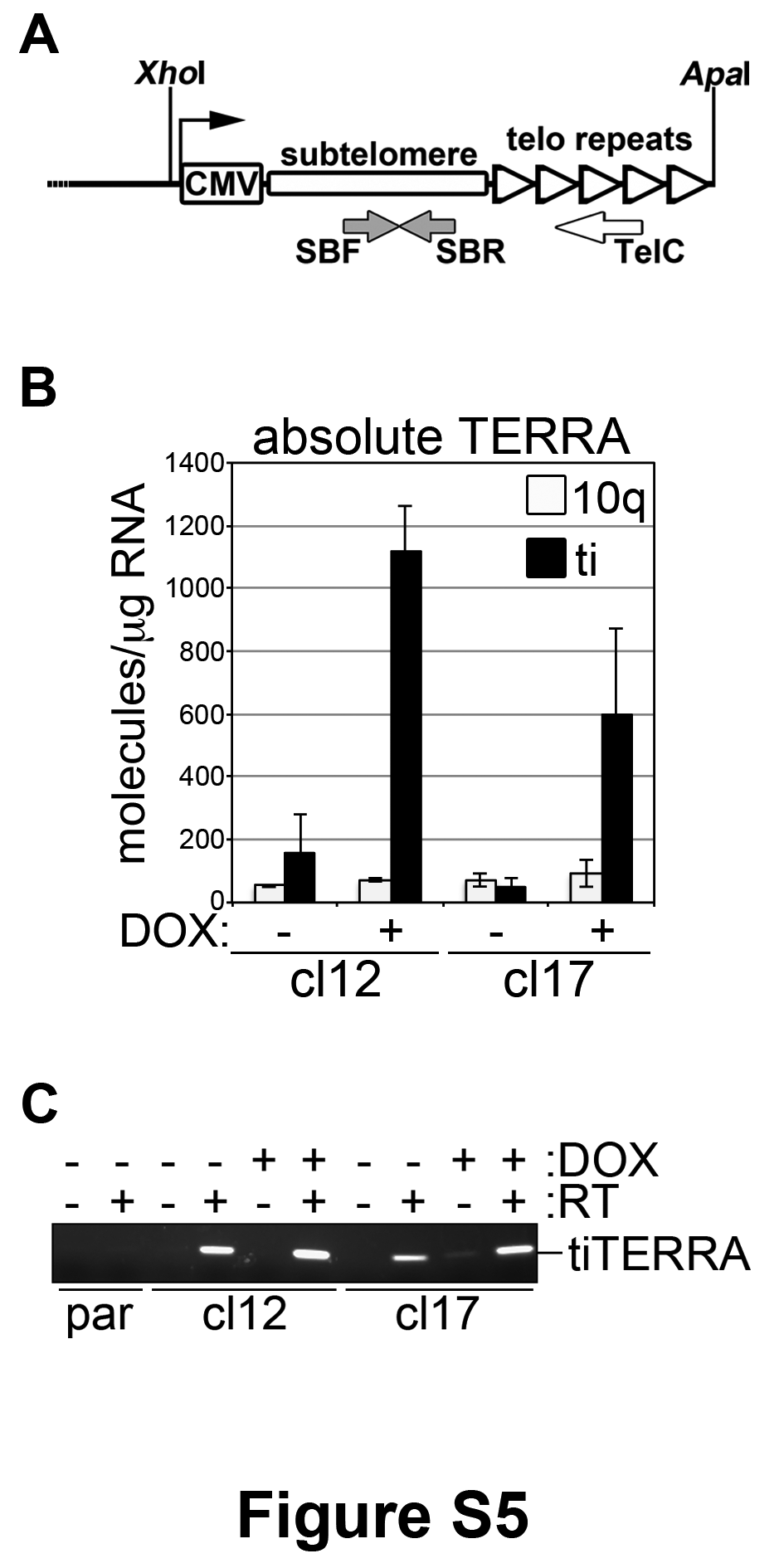

Supplement: Figure S5 — Shows the characterization of tiTEL transcription induction in cl12 and cl17 cells. (A) Sketch of oligonucleotides used in RT-PCR experiments. TelC oligonucleotides were used for RT, SBF and SBR oligonucleotides were used for PCR. (B) Absolute quantification of tiTERRA (ti) and natural TERRA transcribed from 10q chromosome ends in cl12 and cl17 cells treated or not with DOX. (C) Agarose gel analysis of tiTERRA RT-PCR products from cl12 and cl17 cells treated or not with DOX. (TIF) [file pone.0035714.s005.tif]

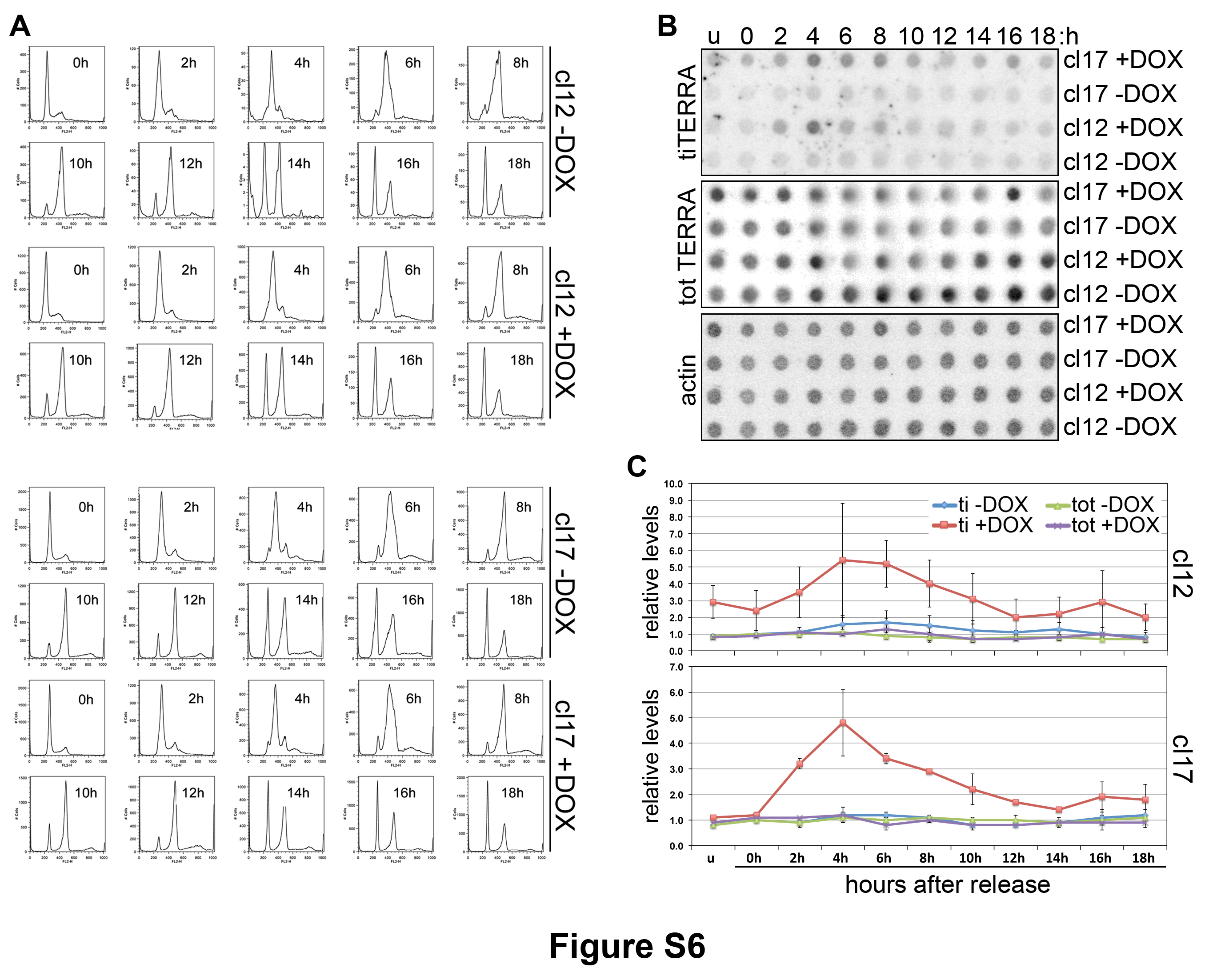

Supplement: Figure S6 — Shows tiTERRA and total TERRA steady-state levels in synchronized cl12 and cl17 cells. (A) FACS profiles of propidium iodine-stained cells blocked in G1/S using aphidicolin and released into the cell cycle for the indicated hours in presence or absence of DOX. (B) Dot blot analysis of total RNA isolated at indicated hours after release. The same membranes were hybridized successively with SBP (to detect tiTERRA), telomeric (to detect total TERRA) and beta-actin (loading control) probes. (C) Quantification of dot-blots as in B. TERRA values were normalized through the corresponding actin values and expressed as fold increase over unsynchronized, untreated cells. Points and error bars represent averages and standard deviations from two independent experiments. (TIF) [file pone.0035714.s006.tif]
