# Supplementary material for: Telomerase Efficiently Elongates Highly Transcribing Telomeres in Human Cancer Cells
Source: PLoS One. 2012 Apr 27;7(4):e35714. doi: 10.1371/journal.pone.0035714 (PMC3338753; doi:10.1371/journal.pone.0035714)
Supplement: Table S1 — Shows the oligonucleotides used in this study. (DOCX) [file pone.0035714.s007.docx]

**Table S1. Oligonucleotides used in this study.**

| **Name** | **Application** | **Sequence** |
| --- | --- | --- |
| Telorette 3 | STELA | 5’-TGCTCCGTGCATCTGGCATCCCTAACC-3’ |
| Telorette 4 | STELA | 5’-TGCTCCGTGCATCTGGCATCCTAACCC-3’ |
| Teltail | STELA | 5’-TGCTCCGTGCATCTGGCATC-3’ |
| tiTel-STELA | STELA | 5’-ACCGGGAGGTAGATGAGATG-3’ |
| Xp/Yp-STELA | STELA | 5’-GTTGTCTCAGGGTCCTAGTG-3’ |
| 15qF | STELA PCR and RT-PCR | 5’-CAGCGAGATTCTCCCAAGCTAAG-3’ |
| TelC | RT-PCR | 5’-[CCCTAA]_5_-3’ |
| SBF | RT-PCR | 5’-AGAATCTCACGCAGGCAGTT-3’ |
| SBR | RT-PCR | 5’-CCAGGGATTTCAGTCGATGT-3’ |
| 10qF | RT-PCR | 5’-GAATCCTGCGCACCGAGAT-3’ |
| 10qR | RT-PCR | 5’-CTGCACTTGAACCCTGCAATAC-3’ |
| U6F | RT-PCR (normalizer) | 5’-GGAATCTAGAACATATACTAAAATTGGAAC-3’ |
| U6R | RT-PCR (normalizer) | 5’-GGAACTCGAGTTTGCGTGTCATCCTTGCGC-3’ |
| 15qR | RT-PCR | 5’-AACCCTAACCACATGAGCAACG-3’ |
| Xp/Yp-F | RT-PCR | 5’-GCAAAGAGTGAAAGAACGAAGCTT-3’ |
| Xp/Yp-R | RT-PCR | 5’-CCCTCTGAAAGTGGACCAATCA-3’ |
| ALU probe | DNA hybridization | 5’-GTGATCCGCCCGCCTCGGCCTCCCAAAGTG-3’ |
| beta-actin | RNA hybridization | 5’-GTGAGGATCTTCATGAGGTAGTCAGTCAGGT-3’ |
| 18S | RNA hybridization | 5’-CCATCCAATCGGTAGTAGCG-3’ |
